# Investigating the amount of macro, meso, and microplastics in the surface soil around the landfill of Tabriz and the effect of the prevailing wind on their distribution

**DOI:** 10.1016/j.heliyon.2025.e42143

**Published:** 2025-01-21

**Authors:** Mohamad Javad Asadi, Mehdi Ghayebzadeh, Seyedeh Maryam Seyed Mousavi, Hassan Taghipour, Hassan Aslani

**Affiliations:** a Department of Environmental Health Engineering, School of Health, Tabriz University of Medical Sciences, Tabriz, Iran; b Department of Environmental Health Engineering, School of Health, Zahedan University of Medical Sciences, Zahedan, Iran; c Infectious Diseases and Tropical Medicine Research Center, Research Institute of Cellular and Molecular Sciences in Infectious Diseases, Zahedan University of Medical Sciences, Zahedan, Iran; d Health and Environment Research Center, School of Public Health, Tabriz University of Medical Sciences, Tabriz, Iran

**Keywords:** Landfill, Plastic, Microplastic, Distribution, Prevailing wind effect

## Abstract

Environmental pollution with plastic and microplastics (MPs) is a global problem. This study investigates macro, meso, and MPs in the soil around the Tabriz landfill in northwest Iran and the effect of prevailing wind on their distribution. One control sample and one sample from the landfill itself, 20 samples in four directions at regular intervals in the direction of the prevailing wind and against it, and two perpendicular directions (22 samples) were taken and analyzed. The results showed that the landfill is poorly managed, and in fact, it is an unsanitary landfill/dump site. The soil around it is polluted with the average abundance of macro, meso, and MPs equal to 6.5 ± 10.4 item/kg(dw), 15.5 ± 28.3 item/kg(dw), and 470 ± 193 item/kg(dw) respectively. The prevailing wind in the region has had no significant effect on the dispersion and distribution of MPs. The most abundant MPs in the soil of the studied area belonged to fragment and film-shaped particles, respectively, and the most abundant color was white. Indiscriminate use of plastics, especially single-use plastics, lack of attention to the hierarchy of waste management, as well as the lack of proper management of the landfill and turning it into a waste dump, are among the most important reasons for the presence of macro, meso, and MPs in the soil of the studied landfill.

## Highlights

• Sampling was carried out from the surface soil around a landfill in Tabriz for analyzing macro, meso, and microplastics.
• The soil was polluted and the average number of macro, meso, and microplastics were about 6.5 ± 10.4, 15.5 ± 28.3, and 470 ± 193 Item/kg (dw).
• The prevailing wind in the region has had no significant effect on the dispersion and distribution of MPs.
• Predominant shape and polymer were fragment and polystyrene, respectively.

## 1 Introduction

With the increasing population and mismanagement of plastic waste, the world and Iran are currently witnessing an increase in the amount of plastic waste, and this issue is known as one of the emerging threats to health and the environment [1,2]. Plastics are present in almost all ecosystems [1,3,4], and after entering the environment, are gradually destroyed, decomposed, and converted into macro (more than 25 mm), meso (5–25 mm), micro (0.1 μm–5 mm), and nano plastic (smaller than 0.1 μm) [5].

Unfortunately, landfilling as a final disposal method is still widely used worldwide. It has been estimated that about 70 % of the MSW produced in the world is sent to landfills or unsanitary sites and waste dumps [6]. On the other hand, about 8–12 % of solid waste consists of plastics and only a small percentage of waste (especially plastics) is recycled [7]. Therefore, landfills still receive a significant amount of plastic waste. It is estimated that about 42 % of the 359 million tons of plastic waste produced in the world in 2018 were transferred to landfills [2]. Accordingly, it can be theoretically assumed that landfills act as a major source of MPs [8]. After sanitary landfilling or unsanitary landfilling/dumping of waste in the environment, a significant amount of plastics is converted into meso and MPs during complex physical, chemical, and biochemical reactions [2,9]. The presence of MPs and nanoplastics in the soil and even the leachate of landfills has been reported [10]. According to scientific reports, many soils are polluted with large amounts of plastic waste (about 50–250 kg per hectare) [11]; these pollutants usually enter the soil directly (through agricultural, industrial, and other daily activities such as mulching, composting, irrigation, and waste disposal) or indirectly (through runoff, atmospheric deposition or wind flow, etc.). They are considered a potential threat to the survival, growth, and reproduction of soil microbiota, and they can also change soil properties and biogeochemical cycles, negatively affecting agriculture and plant productivity. On the other hand, with the increase in the number of MPs in the soil, water sources and the food chain will likely be polluted, which will have health effects on humans [4,[12], [13], [14]]. Some limited studies have been conducted in different regions of the world on evaluating and identifying MPs in the soil and sediments around landfills, as well as agricultural soil and other soil pollution has been reported [15,16].

Considering the health and environmental effects of the entry of MPs into the environment, identifying, quantifying, and determining the main sources of MPs should be prioritized. Many studies have been conducted on the abundance of MPs in the environment, especially in aquatic ecosystems and some of the agricultural soils. Nevertheless, there are rare studies in the world about macro, meso, and MPs in the soil of the landfills and their surroundings, and there are no available studies and reports in this regard in Iran. Furthermore, no available study has been conducted on the effect of wind on the distribution of MPs in the soil around landfills. Therefore, this study was performed to investigate the amounts of macro, meso, and MPs in the soil surrounding the landfill of Tabriz City in the northwest of Iran and the effect of the prevailing wind on their dispersion and distribution, as well as, Identifying their nature.

## 2 Materials and methods

### 2.1 The study area

**Fig. 1:**
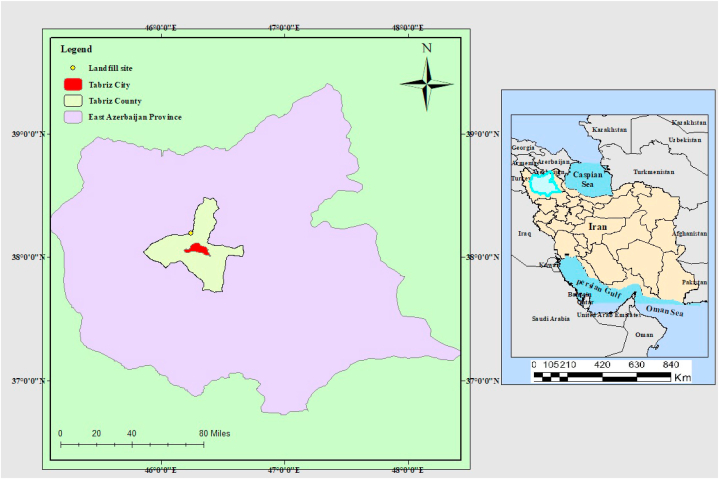
Location of the studied landfill.

This descriptive cross-sectional experimental study was conducted to investigate the level of soil pollution around the landfill of Tabriz city with macro, meso, and MPs in 2023. Tabriz is a city in northwestern Iran and the capital of East Azerbaijan Province, with a population of about 1.7 Million. It is one of the biggest cities in Iran and has an area of 244.51 km^2^ (City boundary: 1014.45 km^2^). Tabriz is located at 38°N latitude and 46°292°E longitude with an average height of 1430 m above sea level and at the extreme east and southeast of Tabriz Plain. This city has a cold and dry climate in winter and hot and dry in summer. The study landfill is located 12 km northwest of Tabriz. The general location of the sampled landfills is presented in Table S1 and Fig. 1.

### 2.2 Sampling

In this research, in the first step, the condition of waste management and disposal in the landfill was documented by collecting information and data and by field observations. In the second step, to determine the amount of macro, meso, and MPs in the soil, in addition to the control sample and a sample from the landfill itself, samples were collected from the soil around the landfill. A stainless steel container (in the form of a rectangular metal frame with an open top and bottom) with dimensions 50 × 50 × 20 cm was used to take samples. Sampling was carried out in four directions of the landfill at regular intervals in the direction of the prevailing wind and against it (to determine the effect of the prevailing wind on the distribution of MPs) and also in two directions perpendicular to the direction of the prevailing wind to the landfill. For sampling from each location, four points were selected at a distance of approximately 100 m, and a homogeneous sample of about 0.5 kg was taken from the surface to a depth of 20 cm. After mixing them, a composite sample weighing about 2 kg was prepared that was considered as a single sample. A total of 22 samples, including 1 sample from the soil of the landfill itself and 5 samples, were taken at distances of 250, 500, 750, 1000, and 1250 m in four directions, in the direction of the prevailing wind and against it (Samples: A_1-5_ were in the direction of the prevailing wind and O_1-5_ were against the prevailing wind of landfill) and also in two directions perpendicular to the prevailing wind (Samples: N_1-5_ are north vertical and S_1-5_ were south vertical of landfill) in May 2023. A control sample was also taken at a distance of about 11 km from the landfill, which was not affected by the landfill. Before sampling, debris such as large stones and other large non-plastic items were separated from the soil. The samples were collected and labeled in aluminum containers and transported to the laboratory.

### 2.3 Preparation of samples

After the completion of sampling, the samples were transferred to the laboratory in the shortest possible time. All samples were dried at laboratory temperature for one week (168 h). Each sample was then wrapped in aluminum foils and stored in a separate, racked room for further analysis.

### 2.4 Extraction of macro, meso and MPs

Identification and evaluation of macro-, meso-, and MPs were done during five main stages: 1. primary screening (only for counting and visual inspection of macro and mesoplastics), 2. separation based on density, 3. digestion, 4. screening and filtration (for classification and counting of small-microplastics (SMPs) and large-microplastics (LMPs), 5. analysis of Raman Spectroscopy and Scanning Electron Microscope (SEM).

#### 2.4.1 Primary screening (for macro and meso plastics)

The dried soil sample of the landfill was mixed in the same aluminum foil with a spoon to obtain a homogenous sample; then, for the analysis of the samples, the larger parts (macro) of the samples were visually examined, and the macroplastics were separated and counted. In the next step, to separate the mesoplastics, the samples were passed through a 4.75 mm sieve (Mesh = 4), and the number of mesoplastics was also counted. Then, 50 g of each sieved sample was taken, and MPs (0.3–4.75 mm) were examined.

#### 2.4.2 MPs processing and analysis

The samples were processed and analyzed using three density separation, digestion, and filtration methods. ZnCl_2_ solution (density: 1.6 g mL^−1^) was used for particle separation and extraction. Digestion was done using H_2_O_2_ (30 %). Filtration was carried out through a cellulose nitrate membrane filter (pore size = 0.45 μm, diameter = 47 mm), by vacuum filtration. Finally, the particles were grouped according to size, shape, and color. Visual and optical analysis of identified particles (determination and classification of particles according to shape, color, and size) was performed using a digital microscope (Model = DM9 Digital Microscope, made in China)). The Micro-Raman spectroscopy method was used to identify the nature of extracted MPs particles (the identified micro Raman spectra were accredited using SLoPP and SLoPP-E Raman spectral libraries for MPs research), and the surface morphology of MPs was determined using scanning electron microscope (SEM), brand FEG-SEM MIRA3. Additional explanations of this section and device specification are provided in the supplementary file (Text S1).

### 2.5 Statistical analysis

For the statistical analysis of the data, two Kolmogorov-Smirnov tests (to check the normality of data distribution) and an ANOVA test were used to check the effect of wind direction and prevailing wind on the distribution of MPs values in the landfill. Next, to compare the samples in different directions with the waste landfill samples and the control sample, the Sample T Test was used.

### 2.6 Quality assurance and quality control

To avoid sample pollution during the entire study process, the laboratory equipment was used of stainless steel and glass. The equipment was thoroughly washed and finally covered with aluminum foil. During the test process and analysis of samples, commute restrictions to the lab were applied, air conditioning and fans were turned off, and doors and windows remained closed. All the analysis process, including preparation, digestion, density separation, filtration, and visual analysis was carried out inside the fume hood [17,18].

To avoid and evaluate the pollution of the laboratory equipment, samples storage place, and pollution arising from commuting and ambient air during the process and analysis, a set of blank control group samples was used. The hot needle examination to affirm the extracted particles were plastic, and to accuracy and validity of the extraction process was implemented recovery tests.

#### 2.6.1 Blank tests

The blank test as a negative control (blank control) was used to avoid and evaluate the pollution of the laboratory equipment and air during the process. For this purpose, according to the reported studies (with slight modifications), 14 distilled water (no MPs) samples were stored in open beakers for 1–14 days in the laboratory and place where the samples were stored. Also, 7 cellulose nitrate filters (0.45 μm) were placed on a glass plate adjoining the microscope, the hood, and the working area [17].

#### 2.6.2 Recovery rate

Recovery rate tests were performed to validate the particle extraction method. Twenty MPs particles as artificial reference particles from 0.3 to 4.75 mm were added to clean sediments (without MPs) weighing 50 g. Samples were extracted using the ZnCl_2_ (1.6 g mL^−1^) solutions. It should be noted that to prepare a clean sediment, first, the sediment sample were heated at 400 °C for 1 h. Then, the samples were digested several times, and the extraction operation was repeated on this soil. These extraction steps were repeated until no impurities (presence of MPs and other organic compounds) were observed in this sediment [17].

#### 2.6.3 Checking accuracy and correctness

In order to check the accuracy of the observer, among the 44 tested samples (including 22 SMPs and 22 LMPs), five samples were randomly selected, and a direct examination of the samples using a microscope was done by three observers. Then, the results were analyzed using the Intraclass Correlation Coefficient test [19].

## 3 Results and discussion

### 3.1 The results of quality assurance, accuracy, and accuracy and recovery rate

The quality control and assurance investigation, the results showed that MPs particles were present in all climate control samples; however, their average number in 14 distilled water and 7 cellulose nitrate filters were 6.35 ± 1.94 and 3.7 ± 1.33, respectively (Table 1). This indicates that there was minimal pollution potential in the laboratory (working environment) during the experiments [20,21]. Checking the accuracy of the measurements showed that the correlation coefficient obtained between the values measured by different observers was less than 0.1. This shows the high precision and accuracy of the measurements [19]. The recovery rate tests showed that 85 % of the MPs particles added to the samples could be identified and measured. This recovery rate is acceptable and indicates the accuracy of the methods used in this study [22].

**Table 1:** Microplastics identified in the blanks.

blank = ultra-pure distilled water samples		blank = cellulose nitrate membrane filter	
Sample	Number of MPs	Sample	Number of MPs
Blank 1	5	Blank 1	5
Blank 2	8	Blank 2	2
Blank 3	3	Blank 3	4
Blank 4	8	Blank 4	5
Blank 5	6	Blank 5	3
Blank 6	9	Blank 6	1
Blank 7	8	Blank 7	4
Blank 8	7	Blank 8	4
Blank 9	9	Blank 9	5
Blank 10	5	Blank 10	4
Blank 11	4	–	–
Blank 12	6	–	–
Blank 13	7	–	–
Blank 14	4	–	–
Mean ± SD	6.35 ± 1.94	Mean ± SD	3.7 ± 1.33

### 3.2 The state of management and operation of the studied landfill

**Fig. 2:**
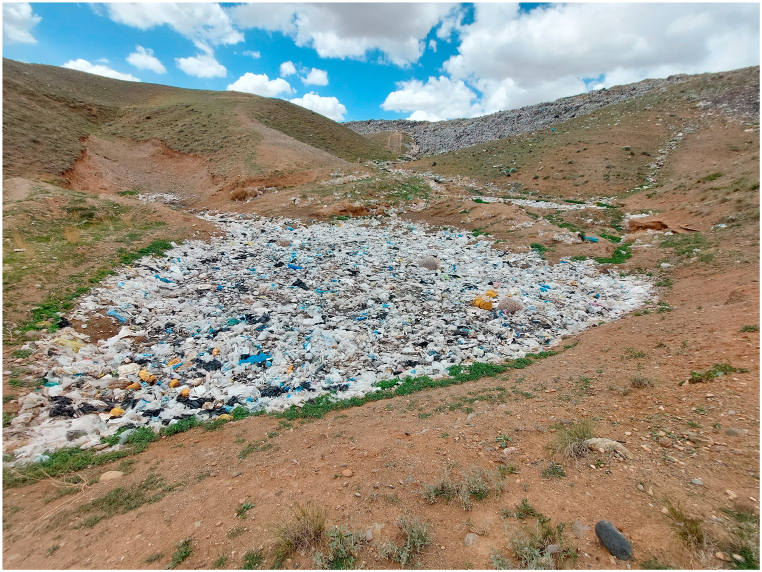
Pictures of the old landfill of Tabriz.

As shown in Fig. 1, the studied landfill is located northwest of Tabriz City. The average rate of municipal solid waste (MSW) production in Tabriz city is about 0.716 kg per person per day (261 kg per person per year). About 1200 tons of MSW (438,000 tons per year) are collected from Tabriz city and disposed of in this center. About 8.25 % of the waste entering the disposal site is made up of plastic [23]. The current landfill does not have the characteristics of a sanitary landfill, and the waste is disposed of in an unsanitary/wasteful and undesirable manner (Fig. 2). A new sanitary landfill nearby is under construction and is not yet be fully operational.

### 3.3 The abundance of macro and mesoplastics in the soil around the studied landfill

The summary of the results obtained from the investigation of the abundance of macro and mesoplastics in the landfill is presented in Table 2. The average abundance of macro and mesoplastics in the total soil samples of the studied landfill was about 2.95 ± 12.62 and 1.24 ± 4.79 item/kg(dw), respectively. The presence of macroplastics and their environmental and health significance have been reported in a UK study (2023) by Billings et al. in the soil of a waste landfill and its surroundings [16]. In a study conducted by Wang et al. (2023), in the soil of agricultural land in China, the average abundance of macroplastics item (kg/ha(dw)) was determined to be 103 [24]. Another study conducted on horticultural soil in China (2022) showed that the average macroplastics in this soil was 139.10 ± 10.03 item/kg(dw). It has been reported that these macroplastics were probably the remains of agricultural activities and mulching [25]. In a study by Gui et al. (2021) in two compost factories in China (Chisong and Licheng cities), the presence of macroplastics in the produced fertilizer was confirmed due to the lack of control of the entry of plastic compounds [22]. In the study of Shekohian et al., in 2021, which was conducted in Iran, the average abundance of mesoplastics in the soil (0–3 and 3–6 cm) in the residential area adjacent to the waste disposal site in Tehran has been reported to be equal to 3.55 ± 1.09 item/kg(dw) and item/kg(dw) 5.25 ± 2.91 respectively [26], which is higher than the values obtained in the present study. In another study conducted by Shekohian et al., in 2023, the average abundance of mesoplastic in the soil (3-0 and 6-3 cm) in the Kehrizak waste landfill in Tehran was reported to be 29.8 ± 4.06 item/kg(dw) and18.1 ± 8.30 item/kg (dw) respectively [27], which again is higher than the values obtained from the present study. The reason for this difference in abundance can be due to the influence of different environmental conditions, such as the wind direction (west to east) in the Kahrizak waste landfill and also the difference in the management of the studied landfill. In the study of Nurhasanah et al. in Indonesia (Galuga City) (2021), the presence of mesoplastics in the amount of 51.11 ± 4.71 item/m^3^ was reported in the waste landfill leachate [28]. The presence of macroplastics and mesoplastics in the soil of landfills can be related to the excessive consumption of plastics (especially single-use plastics and plastic bags), failure to separate and recycle them, as well as their unprincipled and unsanitary disposal in landfills [29]. Entry and existence of macroplastics and mesoplastics can be considered the main source and cause of soil and water pollution around landfills with MPs and nanoplastics [30].

**Table 2:** The results of examining the abundance of macro and mesoplastics in the surface soil around studied landfill.

Sampling points^a^	Macroplastic (Item/kg_(dw)_)	Mesoplastic (Item/kg_(dw)_)
Landfill	58	22
A_4_	2	2
N_1_	1	2
N_2_	1	0
Minimum	1	0
Maximum	58	22
Average	2.95	1.24
standard deviation	12.62	4.79

a No amount of macro and mesoplastics was found in the rest of the tested samples.

### 3.4 Abundance of MPs in the soil around the studied landfill

The results of MPs abundance are presented in Table 3. The average abundance of MPs in the soil around the landfill for SMPs (0.3–1 mm) and LMPs (1.01–4.75 mm) was obtained at 209 ± 82 and 261 ± 163 item/kg(dw), respectively. The average abundance of the total MPs was about 470 ± 188 item/kg(dw). The reason for the high existence of MPs particles in the present study is inadequate waste management (lack of plastic recycling), unsanitary landfill and waste dumping, the presence of scavengers at the landfill, and unprincipled waste recycling. In one study in Iran (2021), the average of MPs abundance in the soil of the residential area adjacent to the waste disposal site of Tehran, in the top 3 cm of soil was 76 ± 34.98 item/kg(dw) and depth 3–6 cm 24.7 ± 19.79 item/kg(dw). The main factor of this abundance was the presence of secondary MPs in the landfill [26]. The results of a study in China (2022) showed that MPs abundance in agricultural soil was variable from 280 to 2360 item/kg(dw). Agricultural activities were reported as the main cause of pollution, and the contribution of the landfill site to the MPs pollution of the soil in this area was relatively low, which indicated the proper functioning of the waste disposal system at that location [13]. The abundance of MPs in the landfill soil in China (2022) varied from 570 to 14200 item/kg(dw). The main causes of this pollution are the weathering of buried plastic waste and poor waste management [31]. Investigation of the existence of MPs in a landfill and a park in Brazil (2022) showed that the abundance of MPs in the landfill soil was 2393 items/kg(dw), and park sediments were 1401 item/kg(dw). The presence of MPs in park sediments, usually a clean and pollution-free place, indicates the extent of MPs pollution in the environment and the need to increase public awareness about this environmental problem [32]. Various studies have shown that the type of land use and the discharge of runoff, wastewater, and solid waste affect the abundance of MPs particles in soil and sediments [12,28,33,34]. It has also been reported that inadequate management of waste and plastics, and their lack of recycling contributes to plastic and MPs distribution and dispersion [[35], [36], [37]]. In the present study, about 44.5 % of the MPs particles in the soil around the studied landfill were SMPS, and 55.5 % of them were LMPS, which shows that the abundance of LMPS in the soil of the waste landfill is higher than that of SMPS. This is neither consistent with the results reported in Wan's study on the pollution of waste landfills with MPs [31] nor with another study conducted by Zhou et al. in agricultural soil in Beijing city in which the abundance values of SMPS were reported to be equal to 70 % [38]. Several factors can be effective for the greater abundance of LMPS in the soil of the waste landfill compared to SMPS: 1- LMPS are less likely to be moved by wind or water due to their larger size and weight. As a result, LMPS is likely to remain in landfills and accumulate in the soil. In contrast, SMPS, due to their smaller size and weight, are easily moved by wind or water and can be transported from landfills to other locations. Also, SMPs are more easily degraded due to their higher surface area/volume ratio, affecting their faster transfer and movement [39].

**Table 3:** Abundance and results of descriptive statistics of MPs in the soil of Tabriz landfill site.

Sampling points		Sampling depth (cm)		
		0–20		
		SMPs (Item/kg_(dw)_)	LMPs (Item/kg_(dw)_)	MPs = (SMPs + LMPs) (Item/kg_(dw)_)
Agree with the prevailing wind	A_1_	180	300	480
	A_2_	200	200	400
	A_3_	120	460	580
	A_4_	140	300	440
	A_5_	140	240	380
North vertical	N_1_	240	360	600
	N_2_	280	820	110
	N_3_	120	260	380
	N_4_	220	180	400
	N_5_	480	100	580
South vertical	S_1_	160	100	260
	S_2_	260	440	700
	S_3_	320	300	620
	S_4_	200	220	420
	S_5_	200	160	360
Against the prevailing wind	O_1_	200	200	400
	O_2_	180	100	280
	O_3_	160	160	320
	O_4_	120	140	260
	O_5_	260	180	440
Example of landfill (L_1_)		1820	6040	7860
Control sample (B _1_)		60	120	180
Minimum		120	100	260
Maximum		480	820	1100
Average		209	261	470
Standard deviation		82	163	188

1 = L and B samples are not calculated in the descriptive statistics.

The results of a one-way analysis of variance to compare the distribution of MPs in the direction of the prevailing wind and against, as well as in two directions perpendicular to the prevailing wind, showed that in the different sampled directions there is no significant difference between the number and also the distribution of SMPs and LMPs (SMPs, Pv = 0.166) and (LMPs, Pv = 0.339). This means that the number and dispersion of MPs in different directions sampled in the landfill were not affected by wind and prevailing wind.

The comparison of the amount of MPs in the studied samples in the soil around the landfill with the soil of the control sample taken at a distance of 11 km from the landfill and also the soil of the landfill itself is presented in Fig. S1. As can be seen in Fig. S1 and Table 3, the amounts of SMPs and LMPs in the control sample soil are 60 item/kg(dw), and 120 item/kg(dw), respectively. The sum of large and small MPs was equal to 180 item/kg(dw), which was much lower than the average values observed in the soil samples around the landfill 470 item/kg(dw) and the soil of the landfill itself 7860 item/kg(dw). Statistical analysis showed that the number of MPs in the soil of the landfill was different compared to the control sample, and this difference was significant (Pv > 0.001). This shows that the soil around the landfill has been polluted with MPs due to the lack of proper management and exploitation of the landfill, turning it into a dumping site for waste, especially plastic waste, over the past few decades. It has been reported in previous studies by other researchers that due to improper management and disposal, plastic waste in landfills is gradually broken into smaller pieces under the influence of environmental conditions such as ultraviolet (UV) radiation, weathering, and hydrolysis, and over time Their values increase [[40], [41], [42], [43], [44], [45]].

### 3.5 Analyzing the nature of microplastics in terms of their appearance, color, polymer type, and morphology in the studied landfill

#### 3.5.1 Examining the nature of microplastics in terms of appearance and color

**Fig. 3:**
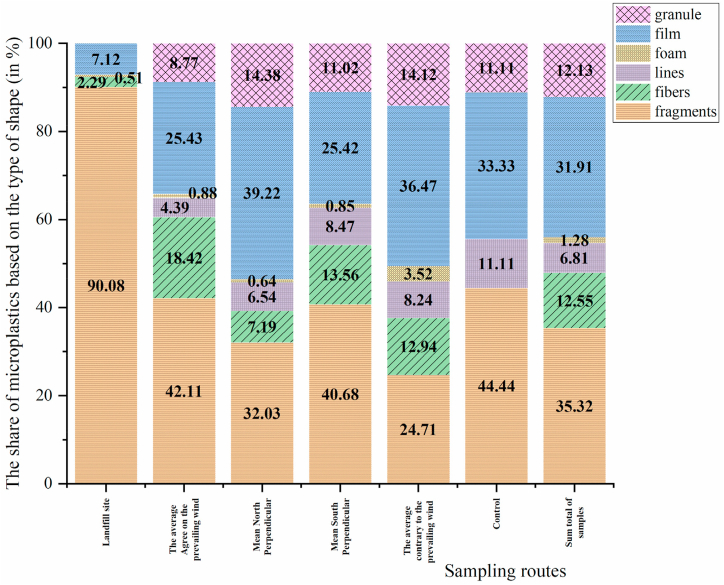
Amounts of MPs based on their shape in the sampled routes and the control sample.

The typical MP shapes are presented in Fig. 3. The types of MPs observed in the present study were fragment (32.4 %), film (31.9 %), fiber (12.5 %), granule (12.1 %), lines (6.8 %), and foam (1.3 %). The results of various studies have reported that fragment-shaped particles have a high abundance [1,8,46]. One of the reasons for this frequency is the increase in the amount of plastic waste and the lifespan of plastic products in landfills [6]. The main source of fragments is the existence of larger plastic debris. The fragments are mainly originated from fragmented hard plastics and have irregular shapes [1,8,46].

Film-shaped particles in terms of abundance (31.9 %) in the landfill soils were in second place. The results of the present study are consistent with some studies [35,47]. The main reasons for the presence of film-shaped MPs in the landfill soil are probably due to the entry of plastic waste from packaging and solid plastic materials, destruction and fragmentation of larger plastic waste such as bottles, plastic bags, etc. Plastic bags are easy to transport due to their lightweight, so their dispersion in the soil around the landfill is high [58,59]. Film-shaped MPs in the soil increase the rate of water evaporation from the soil due to the creation of pores [60]. Therefore, they affect the water distribution in the soil and even increase the pH of the soil [61]. However, it has been reported that fragmented MPs can also affect soil porosity [62]. The main source of fiber and line MPs in landfill soils is unmanaged textiles in MSW [48].

**Fig. 4:**
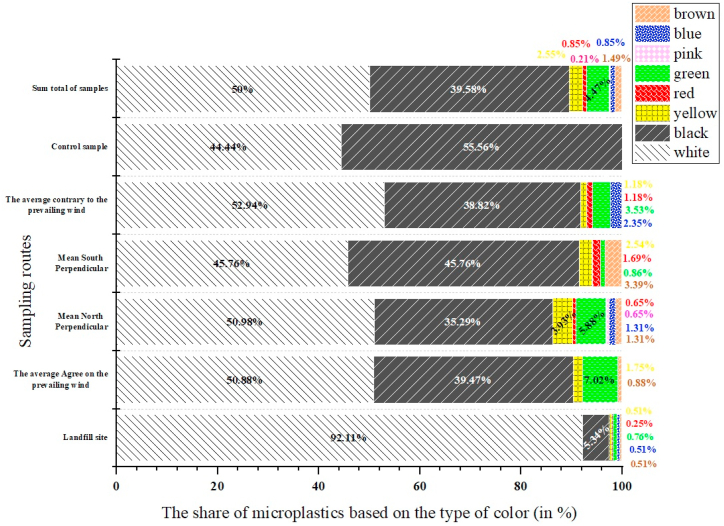
Amounts of MPs based on their color in the sampled routes and the control sample.

The colors of the identified MPs are presented in Fig. 4. The main colors of MPs identified were white (50 %), black (39.6 %), green (4.4 %), and yellow (2.5 %), and the partial values were related to red, pink, blue and brown colors. Other studies report confirmation of the high abundance of white/transparent and black particles in the landfill sediments [[49], [50], [51], [52]]. There is no evidence that the age of the landfill and leachate or H_2_O_2_ used for the acid digestion stage of organic matter has caused color changes in MPs [53]. The reason for the predominance of white color in the MPs in the soil can be related to their environmental degradation process. When MPs are exposed to environmental factors such as sunlight, oxygen, and microorganisms, the pigments in them break down and disappear. Finally, this degradation process results in white MPs particles remaining [67].

Also, it can be expressed that colors may be the factor that interactively affects plastic aging and MP formation [54]. Each polymer has different chemical properties, affecting its resistance to various substances. The UV radiation, oxidizing agents (e.g., chlorine bleach), organic solvents (e.g., acetone), temperature, and additives (e.g., plasticizers, flame re-tardants) affect MPs chemical behavior. Hence, MPs may change color and become transparent during digestion [54].

In the study of Mohammadi et al. (2022), by examining raw leachate samples from Behshahr city in Iran, they reported that the predominant color of MPs was black (45.9 %) and white/transparent (33.4 %). The high frequency of black color in this study is attributed to the disposal of black plastic bags in landfills [52]. In the study by Li et al. in China (2022) in the soil of a sanitary landfill and an unsanitary waste landfill [51] and the study by Piehl et al. in Germany in the soil of agricultural land (2018), white color was determined as the dominant color. It is necessary to explain that the reason for the predominance of white color in the MPs in the soil can be related to their environmental degradation process. When microplastics are exposed to environmental factors such as sunlight, oxygen, and microorganisms, the pigments in them break down and disappear. Finally, this degradation process results in white MPs particles remaining [50]. Of course, in another study by Rahmani et al. (2023), the dominant color of MPs was reported to be black in the soil of the waste landfill of Hamadan city in Iran [49]. In the study of Natsir et al. (2021) in a waste landfill in Indonesia, they reported blue color as the dominant color of their study [55]. Liu and his colleagues in China (2023) in the soil of the waste landfill, as in the present study, have reported the presence of MPs in white, blue, black, red, yellow, and green colors that white color (58.20 %) was the most frequent [13]. Clear MPs generally originate from single-use plastics such as plastic bags or cups, while colored MPs are mainly secondary MPs and result from the fragmentation of colored MPs [56]. Of course, it is necessary to explain that the color of MPs cannot be used to determine their origin and also to identify potential pollution in the environment because the color of MPs may change during the sampling process and also due to environmental conditions. For this reason, spectroscopic and chemical analyses are usually used to identify MPs [56,57].

#### 3.5.2 Analysis of MPs based on the type of polymer

**Fig. 5:**
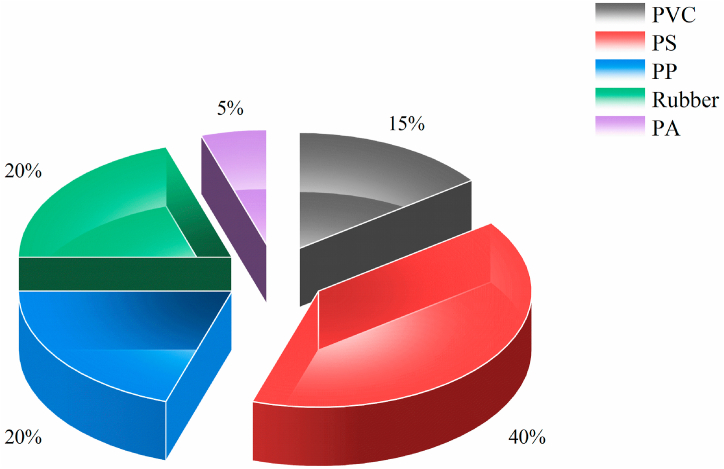
The frequency of the types of polymers identified in the landfill under study.

Micro-Raman spectra are presented in Fig. S2. Polymer-type distribution trends in different samples, are demonstrated in Fig. 5. Identified polymers in the study were (PS = 40 %), (PP and Rubber 20 % each), (PVC = 15 %) and PA = 5 %, respectively. PS particles in the soil may be due to the use of plastic cups for drinks, thermal insulation, and food containers and the disposal of waste attributed to them [58]. The origin of PP particles in the landfill soil can be determined by the use of packaging containers for dairy products, diapers (baby, adult), packaging films, plastic containers, and plastic bags [58]. The presence of rubber particles in the soil can be due to the presence of tires left in landfills and the use of hoses, gaskets, seals, artificial leather, covers, cable covers, transmission belts, etc. [59,60]. The presence of PVC particles in the landfill soil can also be caused by window frames, electronics bodies, bottles, packaging, plastic containers and tanks, plastics used in plumbing and sewage pipes, floor coverings, and furniture [[58], [59], [60], [61]]. PA particles in soil may be due to the use of synthetic fabrics, automotive industry, fishing equipment, carpets, packaging, etc. [59]. Polyethylene (PE) is known as one of the most common polymers in the environment. The widespread presence of this polymer in environmental samples is also mainly associated with the widespread use of plastic products, including plastic bags, plastic bottles, food storage containers, and disposable packaging containers. However, this polyethylene (PE) was not detected in the examined soil samples in the present study. This issue is likely to be due to the recycling of PE during the waste collection process by the garbage collectors and with the people themselves and their less entry into the study landfill. Based on this, the MPs detected in the removed sample are generally related to plastics that are not recycled or are less recycled [62,63]. In the study of Chamanee et al. (2024), the presence of PS and PP polymers in the soil of Sri Lanka's waste landfill has been confirmed, as in the present study [64]. In two studies conducted by Canopoli et al. (2020) and Yu et al. (2022), the presence of both PP and PE polymers was reported in landfills in England and China, respectively [65,66]. Also, in the study of Kazour et al. (2019), the presence of polymers such as PS and PP in an abandoned waste landfill in France has been confirmed [67]. Prague et al. also identified PS and PP, PVC, and PA polymers in the study of leachate samples from 11 landfills in Finland, Iceland, and Norway [68].

#### 3.5.3 Morphological analysis of the surface of MPs

**Fig. 6:**
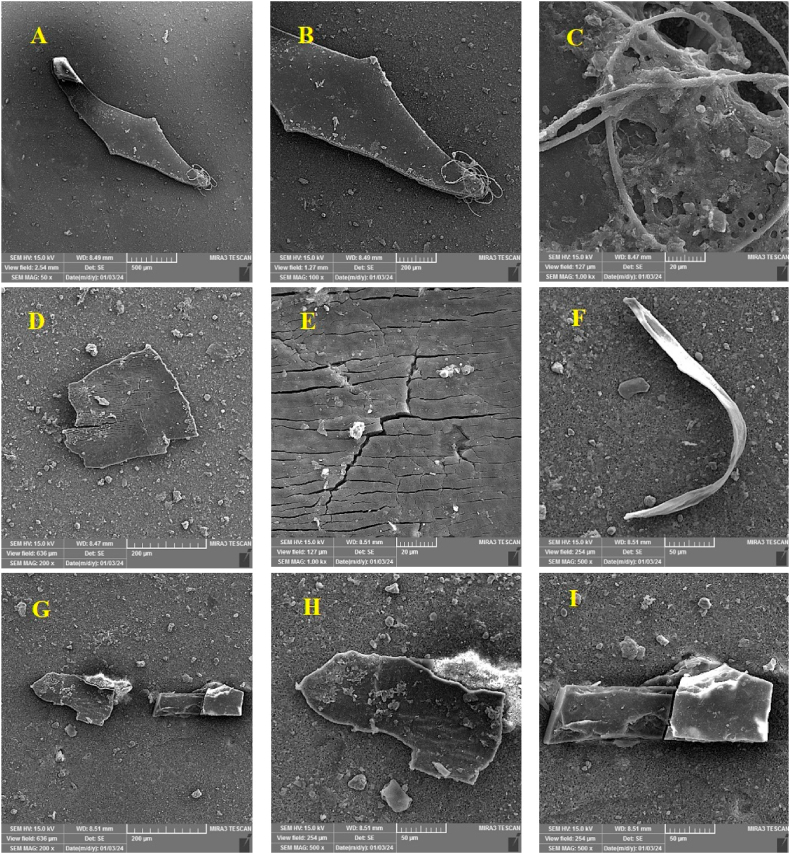
Morphological characteristics of different forms of plastic particles with high-resolution fragments (A, B, D, E, G, H), film (I), fiber (C, F).

Fig. 6 shows the analysis and investigation of the surface morphology of MPs using SEM. The surfaces of MPs identified in the present study are often smooth or irregular. Surface roughness, including fractures and surface grooves, can be seen on several particles. So that uneven pores, porosity, and large and small holes can be seen on the surface of some particles. Also, some particles had worn and broken surfaces, and some had smooth surfaces with sharp or uneven edges. Based on the results obtained from the present study, the surfaces of the piece-shaped MPs have relatively smooth surfaces with irregular and sharp edges. Film-shaped MPs have roughness and surface characteristics such as roughness, cracks, and grooves. In previous studies by other researchers, it has been reported that angular MPs with sharp edges probably entered the environment recently. In contrast, those with smooth edges have been present in the environment for a more extended period [69,70]. These morphological characteristics show that MPs in the waste landfill are probably affected by physical/chemical weathering, mechanical breakage, and long-term oxidation in the environment. This means that the MPs detected in the soil of the studied area were probably caused by secondary MPs, and the lifetime of these plastics in the landfill is longer and they have been degraded during this time and the degradation continues. However, some of these particles were also detected without any signs of weathering. It can be inferred that these particles have recently entered the environment, and during this period, mechanical destruction, abrasion, and weathering of these particles have occurred less [71]. Some morphological characteristics of the surface of MPs, including the presence of unevenness on their surfaces, roughness, cracks and grooves, porosity, and holes created on the surface of the particles, can probably affect the ability to absorb and transfer various environmental pollutants including organic pollutants (for example POPs and PAHs) and heavy metals [17,72,73].

## 4 Conclusion

According to the study results, the conditions of waste disposal in Tabriz city are unfavorable. The average abundance of macro and mesoplastics in the soil around the landfill was 2.95 ± 12.62 item/kg(dw) and 1.24 ± 4.79 item/kg(dw), respectively. The average abundance of MPs in the soil around the landfill site for SMPs and LMPs is 209 ± 82 item/kg(dw) and 261 ± 163 item/kg(dw), respectively, and the average abundance of total MPs is equal to 470 ± 188 item/kg(dw). A one-way analysis of variance was used to compare the number and dispersion, as well as the distribution of SMPs and LMPs in different sampling directions, but no significant difference was observed. This meant that the dispersion of MPs in the directions sampled in the landfill was not affected by wind and prevailing wind. Also, the results of this study showed that there were six types of MPs in terms of their appearance in the soil, and the most common types of MPs were fragments and film-shaped particles. The most abundant color of MPs in the soil of the studied area were white. Five types of polymers were identified in the soil around the landfill, the highest and lowest frequency of which was PS (40 %) and PA (5 %) respectively. The results of this study suggest that excessive use of plastics, especially single-use plastic bags, lack of attention to the hierarchy of waste management, lack of proper management of the city's landfill and turning it into a dumping site are among the most important causes of the presence of macro, meso, and MPs in the soil of studied landfill.

## CRediT authorship contribution statement

**Mohamad Javad Asadi:** Writing – original draft, Resources, Conceptualization. **Mehdi Ghayebzadeh:** Writing – review & editing, Methodology, Data curation, Conceptualization. **Seyedeh Maryam Seyed Mousavi:** Writing – review & editing, Conceptualization. **Hassan Taghipour:** Writing – original draft, Supervision, Project administration, Methodology, Data curation, Conceptualization. **Hassan Aslani:** Writing – review & editing, Conceptualization.

## Ethical approval

Approval was obtained from the institutional-level Medical Ethics Board of Trustees (MEBoT) at Tabriz University of Medical Sciences (Approval Number: IR.TBZMED.VCR.REC.1402.004).

## Data availability statement

The study data will be available to the global research community upon reasonable request from the corresponding author under the regulations of the funding organization.

## Funding information

This study received partial funding from the 10.13039/501100004366Tabriz University of Medical Sciences, under grant number 71337.

## Declaration of competing interest

The authors declare that they have no known competing financial interests or personal relationships that could have appeared to influence the work reported in this paper.
