# Supplementary material for: Investigating the amount of macro, meso, and microplastics in the surface soil around the landfill of Tabriz and the effect of the prevailing wind on their distribution
Source: Heliyon. 2025 Jan 21;11(2):e42143. doi: 10.1016/j.heliyon.2025.e42143 (PMC11795091; doi:10.1016/j.heliyon.2025.e42143)
Supplement: Multimedia component 1 [file mmc1.docx]

**Supplement**

**Investigating the amount of macro, meso, and microplastics in the surface soil around the landfill of Tabriz and the effect of the prevailing wind on their distribution**

Mohamad Javad Asadi ^1^, Mehdi Ghayebzadeh ^2,3^, Seyedeh Maryam Seyed Mousavi ^1^, Hassan Taghipour ^4*^, Hassan Aslani ^1^

^1^ Department of Environmental Health Engineering, School of Health, Tabriz University of Medical Sciences, Tabriz, Iran

^2^ Department of Environmental Health Engineering, School of Health, Zahedan University of Medical Sciences, Zahedan, Iran.

^3^ Infectious Diseases and Tropical Medicine Research Center, Research Institute of Cellular and Molecular Sciences in Infectious Diseases, Zahedan University of Medical Sciences, Zahedan, Iran

^4*^ Health and Environment Research Center, School of Public Health, Tabriz University of Medical Sciences, Tabriz, Iran.

**Corresponding author.** Dr. Hassan Taghipour, Tabriz University of Medical Sciences, Department of Environmental Health Engineering, School of Health, Tabriz, Iran, **e-mail**: [hteir@yahoo.com](mailto:hteir@yahoo.com)

**Text S1**

- **Extraction of MPs by separation and digestion**

The density of MPs is different depending on the type of polymer, additives in plastics, and substances absorbed into them. In this study, ZnCl_2_ solution (density = 1.6 g mL^-1^) was used for the separation of MPs. The volume of each solution used was one liter. First, cleaned and dried sediments were filtered through a steel sieve with a 4.76 mm pore size (Mesh 4) to segregate possible mesoplastics and other larger debris. Then, 50 g of these filtered sediments, along with the ZnCl_2_ solution (1.6 g mL^-1^) was poured into a beaker with a volume of 2 L, and was shaken at 300 rpm for 30 min. After settling for 24 h, the supernatant was transferred to another beaker. In the following, the above steps were repeated by ZnCl_2_ using two stainless steel sieves Mesh 18 (pore size = 1 mm), Mesh 50 (pore size = 0.3 mm), and two groups of large MPs (LMPs) (1.01–4.75 mm) and small MPs (SMPs) (0.3–1 mm) were separated. (All of the used sieves were model ASTM, E: 11, made of stainless steel by Damavand, Iran).

The samples may contain biogenic organic matter. These materials are often confused with MPs and create difficulties in optically distinguishing plastics from other organic matter. Therefore, a simple digestion method is needed to reduce biogenic organic matter without affecting the polymer's structure. The digestion of biogenic organic matter is different depending on the amount in the samples. In this study, the digest of possible biogenic organic matter in the samples was performed by H_2_O_2_ 30% (30 mL, 55 °C, for 24 h). If needed, more H_2_O_2_ (30%) was added until no natural organic material was visible. MPs were rinsed with deionized water before optical and instrumental analysis. In case sample storage was required, hence, all samples were kept in glass containers containing an isopropyl solution of 70% [21]. Optical analysis of MPs was performed for small suspected particles using an optics digital microscope (Model=DM9 Digital Microscope, made in China) [22].

- **Raman microspectroscopy method and electron microscope analysis (SEM)**

One of the most reliable methods to determine the chemical structure of MP particles and the characterization of chemical species is Raman spectroscopy. For analysis of Micro-Raman, samples were sent to the Institute for Color Science & Technology of Tehran, Iran. The micro Raman device in this study had a confocal microscope for microscopic point-by-point analysis and examination of the sample surface. This device had fast imaging with the SWIFT technique (typically 10x faster than conventional Raman imaging), multiple laser wavelengths, EMCCD detection, Raman polarisation, and even Raman-AFM combination, with high sensitivity and power. The identified micro Raman spectra were accredited using SLoPP and SLoPP-E Raman spectral libraries for MPs research. Raman micro-spectroscopy had multi-laser options (532, 638, 785 nm), confocal imaging 0.5 µm XY, and resolution standard > 1.4 cm-1 to identify MPs.

The SEM is a type of electron microscope that produces images of a sample by scanning the surface with a focused beam of electrons. This device has an electron gun at the top of its column that is used to create an electron beam. Also, there is an electron column to converge the electron beam and make the necessary changes in its performance. The electrons interact with atoms in the sample, producing various signals that contain information about the surface topography and composition of the sample. The surface complexity of MPs, evenness, and unevenness of the surface, as well as other surface characteristics, including smoothness and roughness, porosity, crack, grooves on the surface, damage, and abrasion, were determined using SEM images. To provide SEM images, samples were sent to the electron microscope laboratory of Tabriz University, Iran. SEM (brand FEG-SEM MIRA3, made by Tescan Company in the Czech Republic) was applied by that laboratory to analyze the morphology of the MPs and deliver extremely unblemished and high-magnification images of MPs.

| Table S1. Sampling areas and their location on landfill | | | | |
| --- | --- | --- | --- | --- |
| sample code | The name of the sampled place | latitude | Longitude | Height |
| L | The landfill site itself | 38.18795 ^°^ | 46.25912 ^°^ | 1650 m |
| A_1_ | Sample 1 to the east | 38.18604 ^°^ | 46.2621 ^°^ | 1610 m |
| A_2_ | Sample 2 to the east | 38.18648 ^°^ | 46.26579 ^°^ | 1578 m |
| A_3_ | Sample 3 to the east | 38.18724 ^°^ | 46.26783 ^°^ | 1541 m |
| A_4_ | Sample 4 to the east | 38.1862 ^°^ | 46.27053 ^°^ | 1516 m |
| A_5_ | Sample 5 to the east | 38.1862 ^°^ | 46.27274 ^°^ | 1494 m |
| N_1_ | Sample 1 to the north | 38.19191 ^°^ | 46.25496 ^°^ | 1653 m |
| N_2_ | Sample 2 to the north | 38.19362 ^°^ | 46.25486 ^°^ | 1618 m |
| N_3_ | Sample 3 to the north | 38.19535 ^°^ | 46.25442 ^°^ | 1649 m |
| N_4_ | Sample 4 to the north | 38.19725 ^°^ | 46.25614 ^°^ | 1626 m |
| N_5_ | Sample 5 to the north | 38.19881 ^°^ | 46.25518 ^°^ | 1642 m |
| S_1_ | Sample 1 to the south | 38.18752 ^°^ | 46.25105 ^°^ | 1675 m |
| S_2_ | Sample 2 to the south | 38.1867 ^°^ | 46.2523 ^°^ | 1682 m |
| S_3_ | Sample 3 to the south | 38.1841 ^°^ | 46.25581 ^°^ | 1676 m |
| S_4_ | Sample 3 to the south | 38.18413 ^°^ | 46.25727 ^°^ | 1658 m |
| S_5_ | Sample 5 to the south | 38.18312 ^°^ | 46.25925 ^°^ | 1643 m |
| O_1_ | Sample 1 to the west | 38.1864 ^°^ | 46.24566 ^°^ | 1717 m |
| O_2_ | Sample 2 to the west | 38.19063 ^°^ | 46.24387 ^°^ | 1706 m |
| O_3_ | Sample 3 to the west | 38.19261 ^°^ | 46.24235 ^°^ | 1709 m |
| O_4_ | Sample 4 to the west | 38.19466 ^°^ | 46.24102 ^°^ | 1720 m |
| O_5_ | Sample 5 to the west | 38.19718 ^°^ | 46.24116 ^°^ | 1713 m |
| B | Control sample | 38.23257 ^°^ | 46.30857 ^°^ | 1570 m |

| 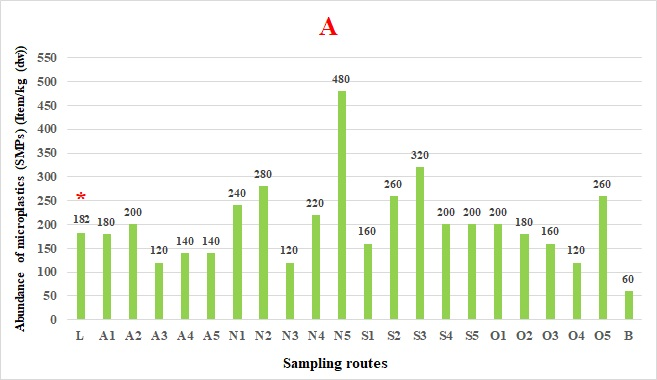 |
| --- |
| 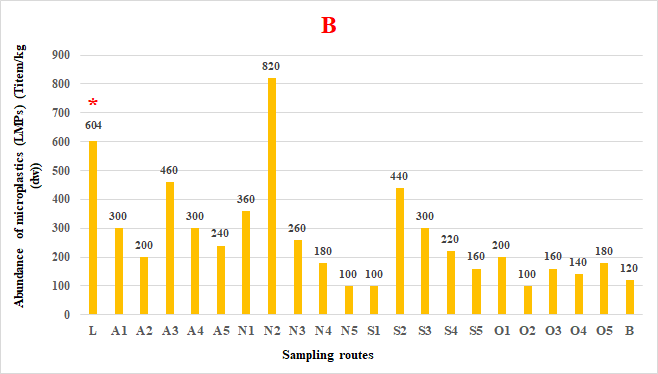 |
| 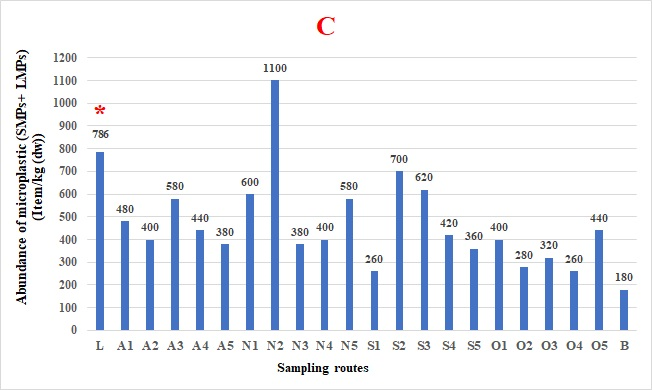 |
| * It should be noted that the scale of the landfill sample values ​​is one-tenth (A = 1820, B = 6040, C = 7860 particles per kilogram of dry soil). |

Fig S1. Comparison of MPs abundance values ​​with each other and the control sample (A = SMPs, B = LMPs, C = (SMPs + LMPs))

| 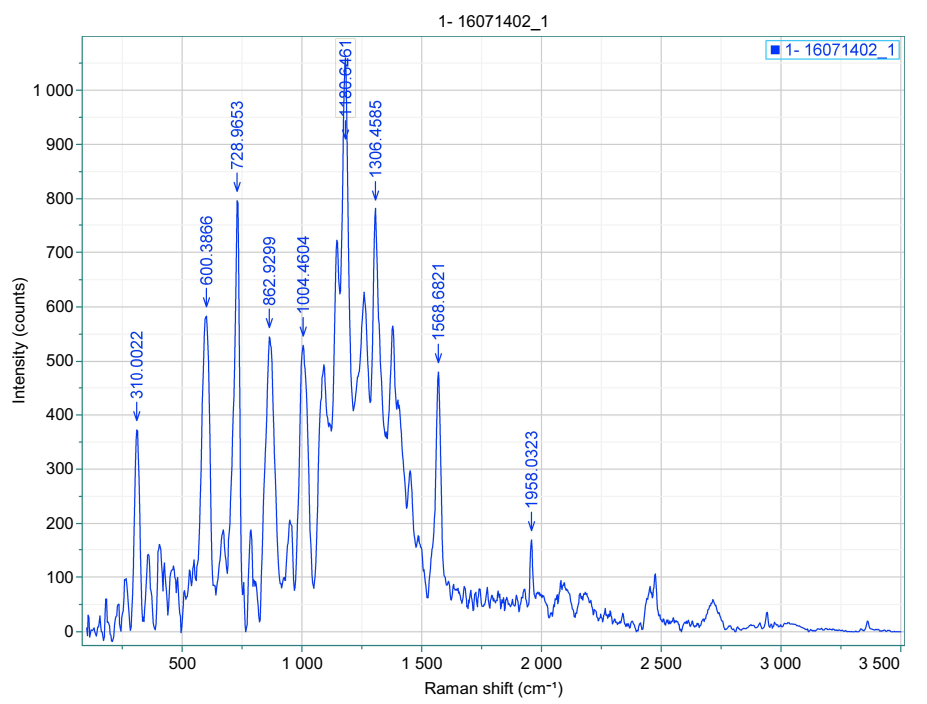 |
| --- |
| **A- (PVC)** |
| 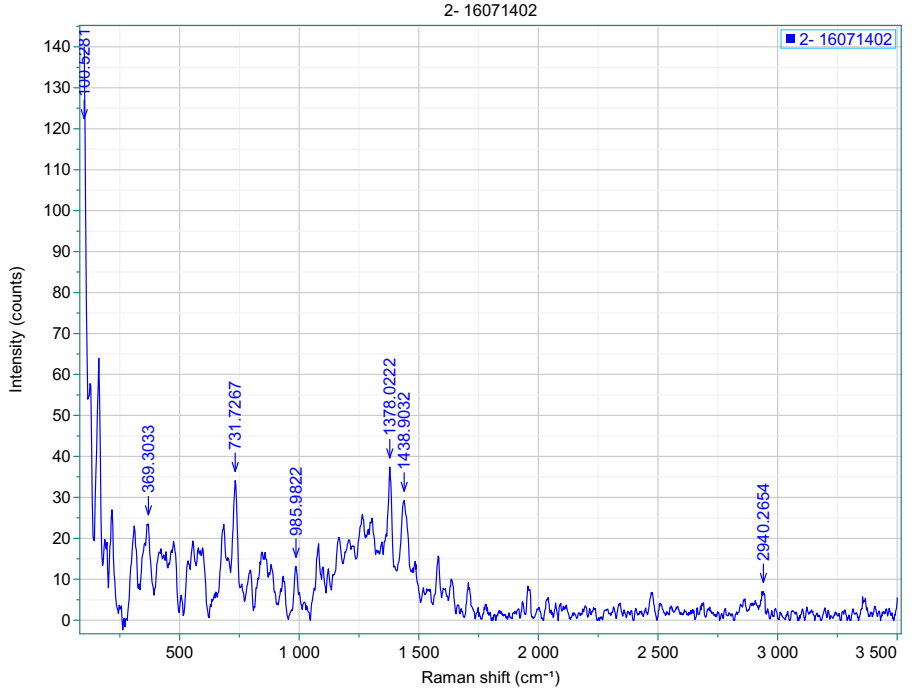 |
| **B- (PS)** |
| 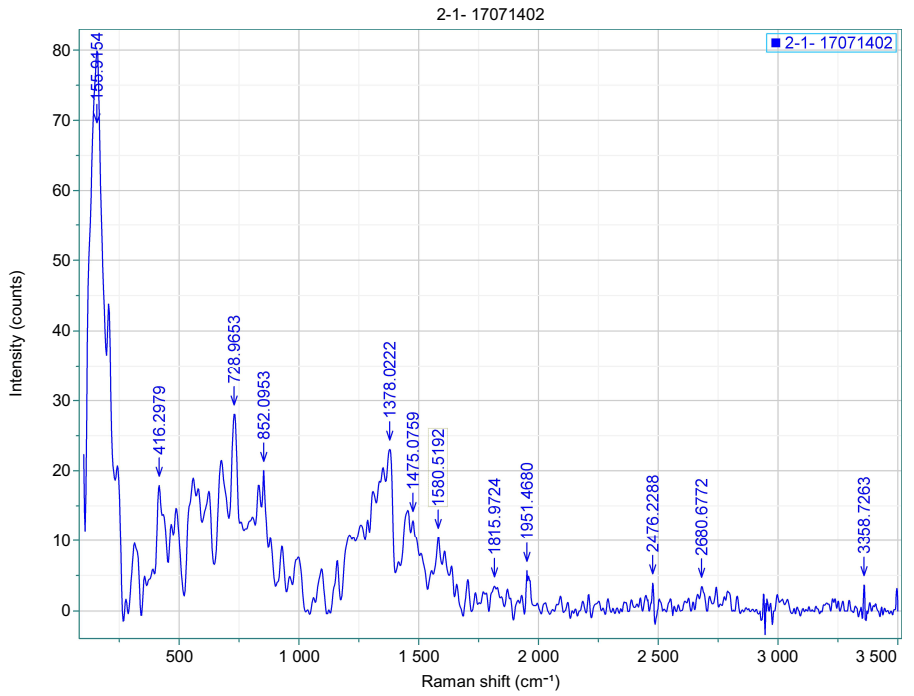 |
| **C- (PP)** |
| 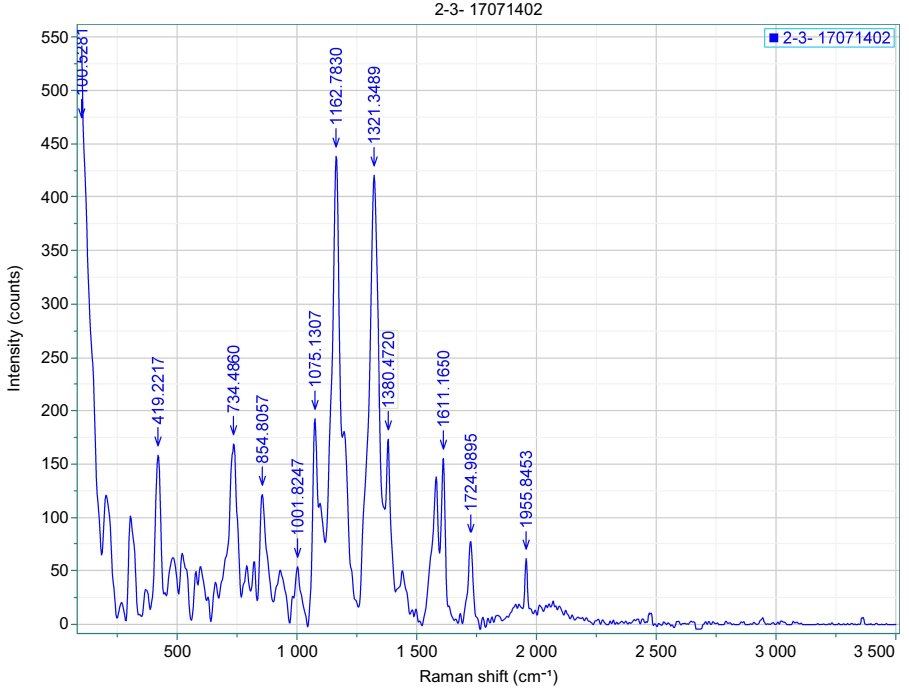 |
| **D- (Rubber)** |
| 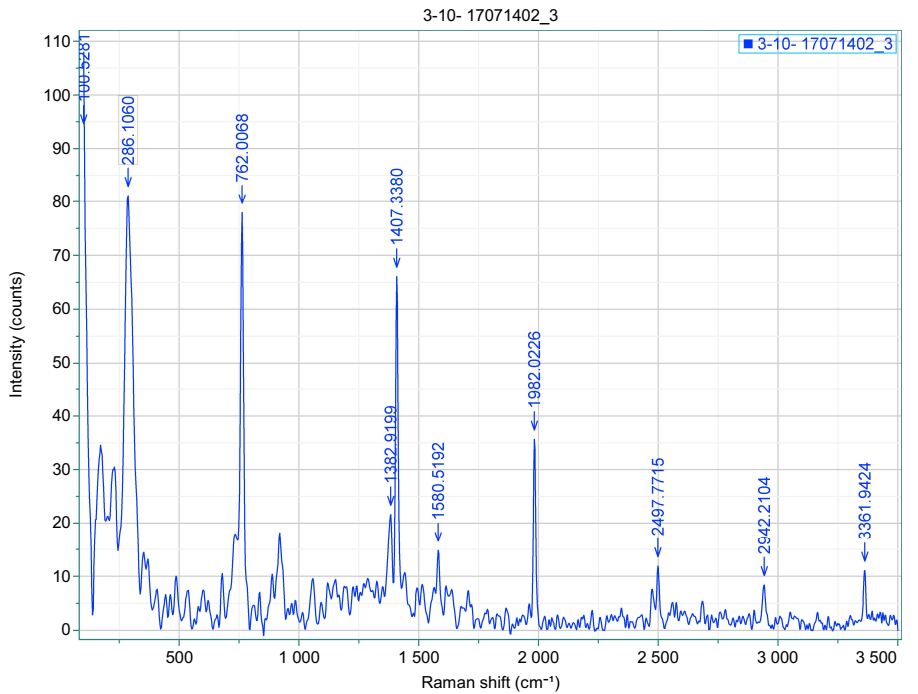 |
| **E- (PA)** |

**Fig S2.** Images show Micro-Raman spectra
